# Social skills development in children aged 4–8 years

**DOI:** 10.1371/journal.pone.0332571

**Published:** 2025-09-26

**Authors:** Krisztián Józsa, Tun Zaw Oo, Diana Borbélyová, Judit Podráczky

**Affiliations:** 1 Institute of Education, University of Szeged, Szeged, Hungary; 2 Institute of Education, Hungarian University of Agriculture and Life Sciences, Kaposvár, Hungary; 3 MTA-MATE Early Childhood Research Group, Kaposvár, Hungary; 4 J. Selye University, Komárno, Slovakia; The University of Manchester, UNITED KINGDOM OF GREAT BRITAIN AND NORTHERN IRELAND

## Abstract

The primary goal of this study was to examine children’s social skills (SS) development by comparing various factors (countries, genders, and age groups). Additionally, this study aimed to analyze how background variables predict the ongoing development of children’s SS. The study involved a total of 3050 Hungarian-speaking children (4–8 years of age) residing in Hungary and Slovakia. First, we investigated measurement invariance (MI) and Latent mean difference (LMD) of the SS assessment test (a part of the DIFER test – Diagnostic Systems for Assessing Development) to assess school readiness in Hungarian preschool children. The findings showed that the SS assessment test is reliable and consistent across different groups of Hungarian preschool children, as it demonstrated MI across all four levels (configural, metric, scalar, and residual) and significant LMD. It was also found that children from Hungary demonstrated superior SS when compared to their counterparts from Slovakia. Furthermore, the analysis of gender revealed that female students exhibited more advanced SS than male students. Additionally, older children displayed significantly higher levels of SS compared to younger children within their respective age groups. Although parental education emerged as a significant predictor of children’s SS development across the entire sample, distinct variations were observed when analyzing each country separately. In both countries, the gender and age of students were identified as highly significant factors contributing to their SS development. Therefore, this study carries substantial importance for educators and researchers alike, offering valuable insights into the assessment and cultivation of students’ SS development.

## Introduction

Assessing children’s school readiness has become increasingly important in many European countries, including Hungary and Slovakia. It is also an important process for preschool children as it helps identify areas where children may need additional support before entering school and can predict academic achievement later on [1]. Many studies [2–4] showed that school readiness assessment is a valuable tool for identifying children who may require additional support in specific areas, which can ultimately lead to better academic outcomes. It also plays a critical role in early childhood education and provides valuable information for educators, policymakers, and families [4]. The purpose of school assessment is to identify any potential areas of weakness and to provide targeted interventions to support children’s academic and social success.

Social skills (SS) are also crucial for the school readiness assessment of preschool children as they play a vital role in their academic and social success [5,6]. SS includes the ability to communicate effectively, work collaboratively, and build positive relationships with others [5]. SS refers to the ability to interact and communicate effectively with others in a variety of social situations. These skills can include things like taking turns, sharing, expressing empathy, following social norms, and constructively resolving conflicts [6]. SS are fundamental abilities that enable individuals to effectively deal with the complexities of interpersonal interactions, providing harmonious relationships and successful integration into society [7]. These SS are also characterized as behaviours that align with social norms and the capacity to engage with others suitably [8]. SS consists of elements like effective social communication, mutual give-and-take in interactions, active engagement in social situations, a readiness to engage with others, and the capacity to establish significant relationships [9].

For children, the development of these skills is of paramount importance, as they serve as building blocks for future social, emotional, and cognitive development [10]. Children with deficits in social competence often struggle with developing and maintaining interpersonal relationships, which can impact their academic achievement [11]. Early childhood is an important time for the growth of SS because even very young children can exhibit age-appropriate capabilities for self-expression and interpersonal communication [8]. Furthermore, one study [2] suggested that when assessing children’s SS development, researchers should scrutinize SS within its contextual framework and take into account the broader setting in which students are expected to demonstrate suitable behaviour. Therefore, understanding the factors that influence the acquisition and progression of SS in children is crucial for educators and researchers to encourage the development of these skills in young children for their school readiness. Various researchers assessed children’s SS development for their school readiness [5,6,12–14].

Recent studies have highlighted the importance of SS for academic achievement, socioemotional development, adaptability to the formal school setting, and mental health outcomes of children [3,15,16]. Therefore, incorporating SS assessment in the school readiness test can provide valuable information for teachers and parents to identify their children’s strengths and areas for improvement. Furthermore, the assessment of SS can help educators in designing developmentally appropriate programs and interventions that support children’s socioemotional development [16]. The assessment of SS can also help in identifying children who may be at risk of developing behavioural or emotional difficulties, thereby allowing for early intervention and support [15]. Assessing SS can also provide valuable information for teachers and parents to better understand children’s behaviour and support their social development [5]. SS research also demonstrated that children’s SS is a strong predictor of academic success. For example, Coelho and Sousa [17] found that students who participated in social and emotional learning programs showed significant improvements in academic achievement. In another study [18] that conducted a meta-analysis of school-based interventions to enhance students’ social and emotional learning, it was found that these interventions/programs led to significant improvements in academic achievement. These findings highlight the importance of SS development in promoting academic success. Therefore, school readiness assessments by SS should be carefully designed and implemented to ensure that they accurately reflect children’s skills and abilities and support their success in later schooling.

Various researchers employed different assessment tools to evaluate children’s SS. For instance, in a particular study [19], researchers utilized Mason Evaluation of SS in Youngsters (MESSY-II), a measurement tool designed for children and adolescents aged 2–16 years. The MESSY-II comprises 64 items, categorized into three distinct factors: Hostile, Adaptive/Appropriate, and Inappropriately assertive. Then, children’s SS was also evaluated using two separate assessments conducted by teachers: one focused on identifying positive social behaviours, and the other aimed at pinpointing problematic behaviours. The evaluation of positive behaviours was assessed by the Personal Maturity Scale and the Social scale rating system. And the assessment of problem behaviour was derived from the Personal Maturity Scale and the Child Behaviour Checklist for Preschool-Age Children-Teacher Report [6]. The development of children’s SS varies across different contexts for various reasons. Differences in social expectations across countries can cause the various cultural norms and shared approaches to caregiving, which impact the timing and methods by which children develop their SS [20]. Another study [3] assessed children’s SS by the use of the Child Behavioural Rating Scale (CBRS) with a five-point Likert scale, rated by teachers. The SS assessment for the school readiness assessment by the DIFER (Diagnostic System for Assessing Development) includes six tests conducted over three separate days: a group task and five individual tasks [1]. SS was also evaluated by employing a German translation of the SS Scale (SSS) from the preschool parental version of the SSRS (SS Rating System). This scale encompasses a total of 39 items, which are further categorized into four subscales (cooperation, assertation, responsibility, and self-control). Participants responded using a 3-point Likert-type scale. Therefore, it becomes evident that while diverse researchers employed varying assessment tools, a recurring theme across their evaluations was the measurement of children’s cooperative behaviors – a crucial aspect in fostering the development of SS.

Numerous factors can influence variations in the development of children’s SS. For example, a study [8] explored differences in the progression of SS in various nations, including Japan and China. Additionally, it explored how demographic factors and parenting practices linked to the development of children’s SS in these two countries. The outcomes revealed that, in comparison to the Chinese participants, the Japanese participants exhibited a notably greater improvement in cooperation skills as they grew older. Nevertheless, theoretical perspectives suggest that disciplinary approaches, including the use or avoidance of corporal punishment, can influence the development of self-control skills across cultural contexts [8]. Another study [21] also showed that older children exhibited expected advantages in the assertion and self-control subscales. However, these differences were not evident in the cooperation subscale.

Gender differences can also impact the development of SS in young children, with school boys often showing higher levels of SS performance compared to school girls [22]. Furthermore, one particular study [22] uncovered gender disparities in SS, with only two factors showing notable differences, possibly linked to the utilization of negative humour styles, specifically involving expressions of disapproval and the tendency to say no and interrupt during interactions. Additionally, its findings indicated that a significant portion of males (one in every three) and females (two in every three) encountered challenges in managing various aspects of humour styles and SS, either in some sections or across multiple aspects. In the study referenced [23], the gender-related effect was primarily attributed to the observation that females attained higher average scores on the assessment of SS in comparison to males.

One study [24] investigated the relationship between child refugees’ SS and their parental education. It was found that higher levels of parental education had a positive impact on a child’s SS development by granting access to psychosocial resources that might not be readily available in households with less-educated parents. This study also concluded that parents’ education levels, highly correlated with their socio-economic status, have highly impacted children’s SS development. Another study [22] also highlighted the significance of parental education in shaping the development of children’s SS. Moreover, this study [25] revealed that the overall level of SS among preschoolers was considered average. The analytical results indicated notable differences in preschoolers’ SS based on maternal and paternal education levels. Specifically, children from high- and medium-income families exhibited stronger SS, potentially attributed to their parents’ capacity to invest more in their children’s upbringing and development, along with the provision of conducive living conditions and environments. These skills not only facilitate positive relationships with peers and adults but also contribute to academic success and mental well-being [22]. Therefore, a comprehensive analysis of SS development in children can shed light on various aspects of their lives and future.

Recent advancements highlight the relevance of mediated and contextual influences on socio-emotional development. For instance, Jiang et al. [26] explored how AI-driven chatbots like Replika elicited digitally mediated empathy, facilitating resilience and psychological well-being during COVID-19, providing a digital parallel to social support mechanisms that might shape children’s socioemotional learning. Similarly, a study [27] also demonstrated that social connectivity and system interactivity, when perceived as beneficial, significantly drove continued engagement with short-video apps, suggesting how interactive environments reinforced social engagement. Exploring mobile social media, another study [27] showed that service quality fostered user identification, belongingness, and satisfaction through emotional attachment, illustrating how supportive contexts cultivated through quality interactions may relate to social skills acquisition. Moreover, children’s depressive mood and their problematic use of mobile apps highly impacted on children’s social skills development [27]. It was because their depression could create problems in their daily use of the mobile app and distract their social skills development. Together, these findings provide valuable information that mediated empathy, environmental interactivity, and emotional attachment shape children’s social functioning. Therefore, these findings also guide the authors to explore how culture, environment, and age influence children’s social skills development.

Recent research has demonstrated the importance of evaluating measurement invariance (MI) to ensure that the instrument is reliable and valid across different groups of individuals. For example, Quirk et al. [28] conducted a comprehensive review of MI in school readiness tests. They found that the MI of school readiness tests across different groups of children, including those from diverse ethnic and cultural backgrounds. The authors noted that MI is essential for ensuring that the test is fair and unbiased, and that it accurately measures the construct being assessed. Similarly, Martín-Puga et al. [29] examined the factorial structure of the Academic Procrastination Scale- Short Form (APS-S) and the measurement invariance across gender and educational levels. The authors also determined possible differences in procrastination across gender, educational levels, and grades. Findings showed that scalar and partial scalar invariance were achieved through gender and educational levels, respectively. Previous research [28–30] has demonstrated that tests with high levels of MI are more reliable and valid and provide fair and unbiased assessments of the construct being assessed. By evaluating MI across different groups and over time, researchers can ensure that school readiness tests are suitable for use with a diverse range of children and that the results are comparable across different groups. Together with the MI assessment, another important assessment of children’s psychometric properties is the latent mean differences (LMD). Investigating LMD is important in MI testing because it can reveal whether the same underlying construct is being measured in the same way across groups or time points. LMD refers to the difference in the means of the latent variables between groups or across time in a longitudinal study when testing for MI. Once the three levels of MI (configural, metric, and scalar) have been established, the LMD can also be detected.

To draw the background to our study, some information is necessary about the preschool education and the school readiness tests, and MI testing and its findings of the Hungarian and Slovakian education systems. In both Hungary and Slovakia, approximately 90% of children attend some form of preschool education before entering primary school. In Hungary, preschool education is mandatory for children aged 3–6 years, while in Slovakia, it is compulsory for children aged 5–6 years [31]. Preschool education in both countries aims to develop children’s social, emotional, cognitive, cognitive, and physical skills through activities such as storytelling, singing, art, physical exercise, and games [32]. These activities intend to help children develop communication, cooperation, problem-solving, and creativity skills. Although Hungary and Slovakia both have some other school readiness tests, such as OVI (Óvodás Vizsgá, kindergarten readiness test) and SOA (School Open to All, screening and diagnostic program) tests for assessing children’s school readiness, the DIFER test (Diagnostic Systems for Assessing Development) has become widely accepted as a reliable and useful assessment test for diagnosing students’ basic skills development [1].

However, there was a lack of studies that have examined the measurement invariance and latent mean differences of the SS assessment from the DIFER test, especially for Hungarian preschool children living in Hungary and Slovakia. While there may be research on MI and LMD in other contexts or with other assessment tools [33], limited research has examined the cultural differences in the SS emphasized in the tests, and how this may impact their validity across different cultural contexts. Therefore, there is a need to investigate whether SS, as measured by the DIFER test, has MI and similar LMD across Hungary and Slovakia. Such research can help educators and policymakers in both countries ensure that school readiness tests are valid and reliable indicators of children’s preparedness for primary school, and help identify areas where improvements are needed to better support children’s development. Therefore, this study first measured the psychometric properties (measurement invariance and latent mean differences) of SS as a school readiness test (DIFER) for Hungarian preschool children in Hungary and Slovakia.

Based on the above review of SS development, it is important to recognize that children’s socialization is a multifaceted process influenced by various factors. These factors may include family dynamics, early educational experiences, cultural norms, and social expectations. Moreover, scrutinizing the similarities and differences in children’s SS development shows how these factors shape their SS development. Given the importance of SS, several researchers have focused on studying their significance in children’s development [14,34,35,36,37]. However, cross-cultural studies examining SS development in Hungary and other countries, such as Slovakia, are scarce. While both nations share a geographic and historical proximity, they also exhibit distinct cultural, social, and educational landscapes. Examining SS development in these countries can offer valuable insights into how cultural and environmental factors may influence this crucial aspect of child development. Therefore, this study aimed to investigate and compare the development of SS in children aged 4–8 years in Slovakia and Hungary.

Then, the following research questions are addressed.

**RQ1:** Are there any significant variations in the SS assessment across countries, gender, and age groups?
**RQ2:** Are there any significant differences in children’s SS development across countries, genders, and age groups?
**RQ3:** What are the predictors for the SS development of children in both countries?

## Methods

### Ethics statement

The study was conducted in accordance with the Declaration of Helsinki and approved by the Institutional Review Board of the Institute of Education, Hungarian University of Agriculture and Life Science (protocol code 1/2022 and date of approval: 20 January 2022). The recruitment period of this study is from 4–31 January 2023. Informed parental consent (written form) was also obtained from all subjects involved in the study through the respective principals.

### Participants

The study gathered necessary data from a cohort of 3050 Hungarian-speaking children aged 4–8 years, originating from Hungary and Slovakia. Among these students, 1609 (52.75%) are from Slovakia and 1441 (47.25%) are from Hungary. The purposive sampling technique was employed in this study. Children in Hungary usually speak only Hungarian (monolinguals), whereas children in Slovakia often know both Hungarian and the Slovak language (bilinguals). Subsequently, the sample was divided into 1641 males (53.87%) and 1409 females (46.13%). The total population was further categorized into five age groups: 4^th^ year (282 students, 9.21%), 5^th^ year (652 students, 21.31%), 6^th^ year (832 students, 27.21%), 7^th^ year (690 students, 22.62%), and 8^th^ year (594 students, 19.65%). Additionally, the study collected data on parental education levels (primary school, middle school, and tertiary education) and socioeconomic status, categorized into low, medium, and high levels, in both countries as part of our background variables.

### Analysis

For this study, the Multi-Group Confirmatory Factor Analysis (MG-CFA) was applied to investigate measurement invariance (MI) across two different groups of preschool children from Hungary and Slovakia. The standards for measuring invariance at each level rely on evaluating the goodness of fit statistics, such as the chi-square test, Comparative Fit Index (CFI), Tucker-Lewis Index (TLI), and Root Mean Square Error of Approximation (RMSEA). In general, if the model fit decreases by more than 0.01 for CFI or more than 0.015 for RMSEA between the configural and metric models, it suggests a lack of metric invariance. Similarly, a decrease in model fit of more than 0.01 for CFI or more than 0.015 for RMSEA between the metric and scalar models indicates a lack of scalar invariance [8]. In the study, various R packages were employed, specifically ‘stats, car, GGally, ggplot2, aov, and geom violin,’ for statistical analyses including t-tests, ANOVA, and regression analyses [38]. Moreover, the Mplus8 (Version 8.4) for conducting path analysis [35], and IBM SPSS Statistics 23.0 were employed to carry out supplementary assessments and descriptive analyses. To assess the adequacy of our model fit for CFA measures, the study also relied on recommended fit indices. These included ensuring that the Chi-square by degrees of freedom (χ2/df < 5) was less than 5, the root mean square of approximation (RMSEA) was less than 0.06, the standardized root mean square residual (SRMR) was less than 0.08, the Tucker-Lewis index (TLI) exceeded 0.90, and the comparative fit index (CFI) was above 0.90 [36]. Adhering to these criteria helped confirm that the model effectively aligns with the observed data and accurately represents the relationships between the variables.

### Instrument and procedure

The assessment of social skills (SS) involved six tests conducted over three separate days: a group task and five individual tasks. This assessment instrument was adopted from the DIFER (Diagnostic System for Assessing Development) school readiness assessment developed by [1,13]. During the group task (social task), four children collaborated on a fine motor skills task, allowing observation of their social attitudes. The children were instructed to copy line drawings, with one child waiting silently after completion. The teacher observed and noted their social interactions without disrupting their work. Individual face-to-face tests were conducted three times in a separate location to evaluate contact behavior. These tests involved brief stories and discussions related to topics such as ‘theft, compassion, violence, harm, and cooperation,’ with scores indicating the development of a basic moral sense. Task performance was evaluated for endurance, emotional attitude, and concentration over three occasions. These assessments, totaling 20 items on a five-point scale (maximum score 120 points or 100%), provide an overview of SS development, focusing on key components relevant to kindergarten and elementary school social life. This instrument had already been validated in our previous study [39] with good internal consistency reliability (Cronbach’s alpha = 0.95), composite reliability (0.94), and average variance extracted (0.51).

### One-group CFA for model testing

**Fig 1:**
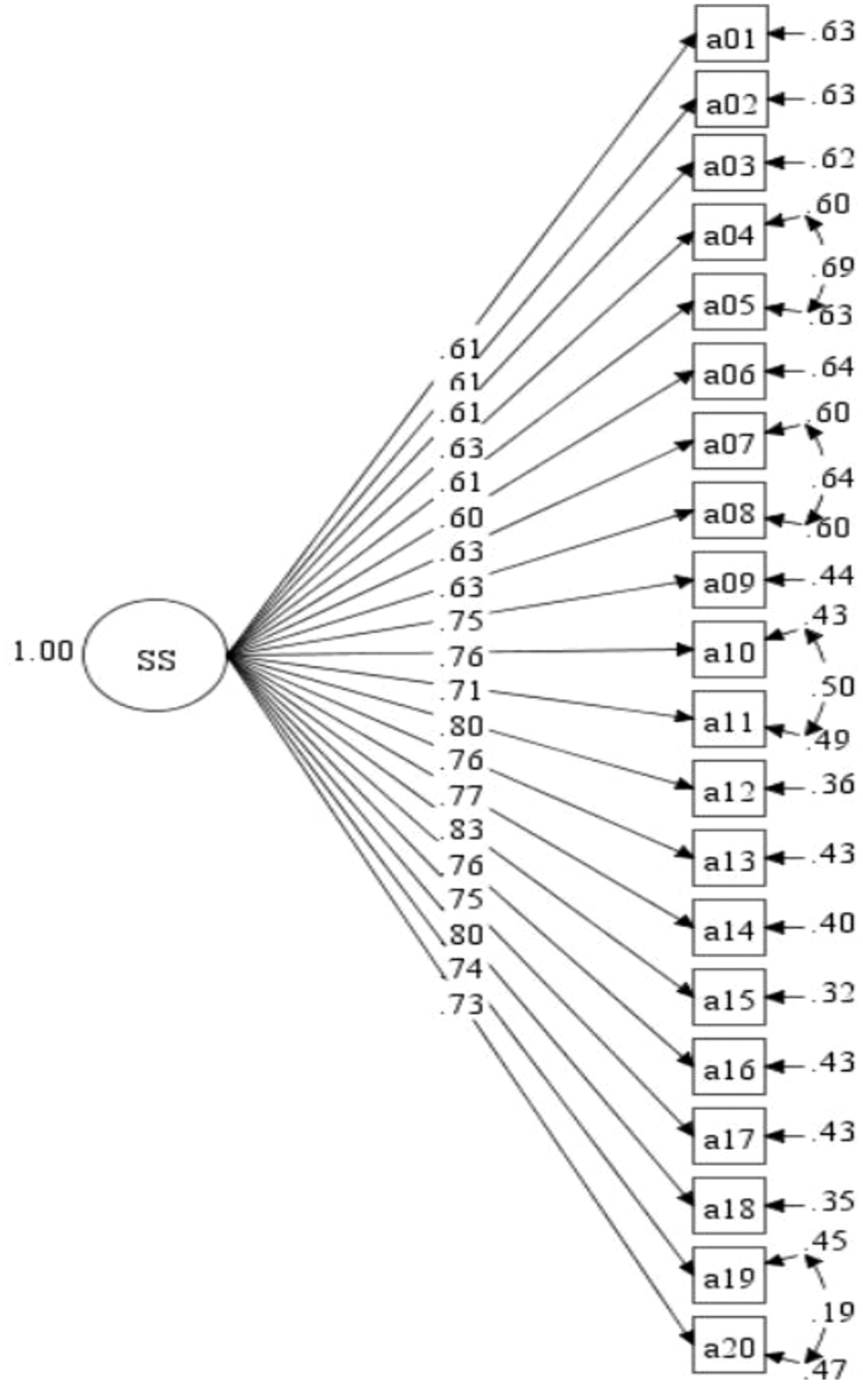
One-group CFA model for the SS assessment.

The study evaluated the SS of young children from Hungary and Slovakia using preliminary analyses. Unfortunately, the fit indices of the original model were not up to our standards (χ2 (df) = 16203.786 (166), CFI = .665, TLI = .623, RMSEA = .127). To refine the model, modification indices were examined, and it was found that certain covariances, such as e04 and e05, e07 and e08, e10 and e11, and e19 and e20, were correlated. After running the modified model, satisfactory fit indices were achieved (χ2/df = 2749.30/144, CFI = .952, TLI = .936, RMSEA = .070), and sufficient factor loadings ranged from.53 to 1.00. The model was considered as the baseline for subsequent MI analyses across various groups on country, gender, and age (Fig 1).

### Multi-group CFA (MG-CFA) for testing measurement invariance

Multi-group confirmatory factor analysis (MG-CFA) was used for testing measurement invariance (MI) and latent mean difference (LMD) of the SS assessment for Hungarian preschool children in Hungary and Slovakia. Testing MI and LMD was divided into three levels: country, gender, and age.

### Measurement invariance across countries

The measurement invariance of the SS assessment was examined across Hungary and Slovakia. The model fit information was evaluated using MG-CFA, and the results showed that the model had a satisfactory fit for both countries (Table 1). Subsequently, measurement invariance tests were conducted, and the results demonstrated that all four invariance models (configural, metric, scalar, and residual) had an adequate fit. Additionally, the ∆CFI and ∆RMSEA values were below the proposed cut-off values (0.01 and 0.015), indicating that a strong invariance across the two countries was supported.

**Table 1:** Measurement invariance across countries.

Model	χ^2^ (df)	CFI (≥0.90)	RMSEA [90% CI]	∆χ^2^	∆df	∆CFI (< 0.01)*	∆RMSEA (<0.015)*	Invariance
**Configural invariance**	3337.6 (288)	.944	.059 [.057,.061]	–	–	–	–	yes
**Metric invariance**	3380.6 (307)	.944	.057 [.056,.059]	43	19	.000	−.002	yes
**Scalar invariance**	3470.1 (326)	.943	.056 [.055,.058]	89.5	19	−.001	−.001	yes
**Residual invariance**	3575.3 (346)	.941	.055 [.054,.057]	105.2	20	−.002	−.001	yes

Note. * (recommended values).

### Measurement invariance across genders

The measurement invariance of the SS of young children was examined across male and female genders. First, the model fit information was evaluated using MG-CFA, and the results showed that the model had a satisfactory fit for both genders (Table 2). Subsequently, measurement invariance tests were conducted, and the results demonstrated that all four invariance models (configural, metric, scalar, and residual) had an adequate fit. Additionally, the ∆CFI and ∆RMSEA values were below the proposed cut-off values (0.01 and 0.015). Therefore, we confirmed that the instrument of young students’ SS assessment had a strong invariance across genders.

**Table 2:** Measurement invariance across genders.

Model	χ^2^ (df)	CFI (≥0.90)	RMSEA [90% CI]	∆χ^2^	∆df	∆CFI (< 0.01)*	∆RMSEA (<0.015)*	Invariance
**Configural invariance**	2953.9 (288)	.950	.055 [.053,.057]	–	–	–	–	Yes
**Metric invariance**	2979.3 (307)	.950	.053 [.052,.055]	25.4	19	.000	−.002	Yes
**Scalar invariance**	3021.4 (326)	.950	.052 [.050,.054]	42.1	19	.000	−.001	Yes
**Residual invariance**	3101.4 (346)	.949	.051 [.049,.053]	80	20	−.001	−.001	Yes

Note. * (recommended values).

### Measurement invariance across age groups

This study was conducted to test the MI of SS in young children across five different age groups (4^th^, 5^th^, 6^th^, 7^th^, and 8^th^ years). We first evaluated the model fit information for each group separately, and the results showed satisfactory model fit for all four age groups (Table 3). Then, the MI was detected across the different age groups. The results showed that the first three invariance models (configural, metric, and scalar) had an adequate model fit. Additionally, the ∆CFI and ∆RMSEA values were less than the recommended values (0.01 and 0.015), indicating a strong invariance across the different age groups at the scalar level. In the residual invariance testing, it was found that the difference in CIF values between residual and scalar models is 0.011, which is more than the recommended value (< 0.01). Therefore, residual invariance of the SS assessment was not achieved at the residual levels. However, despite the lack of residual invariance, the scalar invariance achievement across age groups is enough to accept the subsequent comparisons of SS development across ages [40].

**Table 3:** Measurement invariance across ages.

Model	χ^2^ (df)	CFI (≥0.90)	RMSEA [90% CI]	∆χ^2^	∆df	∆CFI (< 0.01)*	∆RMSEA (<0.015)*	Invariance
**Configural invariance**	3558.2 (720)	.945	.036 [.035,.037]	–	–	–	–	yes
**Metric invariance**	3654.9 (796)	.944	.034 [.033,.035]	96.7	76	−.001	−.001	yes
**Scalar invariance**	3857.8 (872)	.942	.034 [.032,.035]	202.9	76	−.002	.000	yes
**Residual invariance**	4513.9 (955)	.931	.035 [.044,.046]	926.1	83	−.011	.001	No

Note. * (recommended values)

Overall, these findings suggest that the SS assessments used in this study are measuring the same constructs across countries, genders, and age groups, and that the scores can be compared and interpreted in a meaningful way across these groups of countries, genders, and age.

### Latent means difference (LMD) across countries, genders, and age groups

The observed variables’ intercepts were made equal across countries, genders, and ages to compare the latent means of young children. The measurement models in Tables 1–3 had adequate fits for scalar invariance across countries, genders, and ages, indicating that the estimates obtained from this solution are accurate. The results showed that young students from Hungary had significantly higher latent ability (z = 17.886, p < .001) than those from Slovakia, while female students had higher latent ability than males (z = 18.203, p < .001). Additionally, there were significant differences in latent ability among different age groups (4^th^, 5^th^, 6^th^, 7^th^, and 8^th^ years) of young children, with students in higher age groups generally having better latent ability (Table 4). Specifically, 5^th^, 6^th^, 7^th^, and 8^th^-year-old students had better latent ability than 4^th^-year-old students. In another way, students in higher age groups had more latent ability than those in the previous year groups.

**Table 4:** Latent mean differences across countries, genders, and age groups.

Groups	Compared groups	LMD	SE	Critical ratio	Effect size (d)
**Country**	Countries (Slovakia vs. Hungary)	0.01	0.000	20.372***	0.000
**Gender**	Gender (Male vs. Female)	0.15	0.024	20.079***	0.202
**Ages**	4^th^ year vs. 5^th^ year	0.34	0.032	15.153***	0.238
	4^th^ year vs. 6^th^ year	0.50	0.029	16.589***	0.350
	4^th^ year vs. 7^th^ year	0.62	0.029	15.435***	0.444
	4^th^ year vs. 8^th^ year	0.73	0.026	14.428***	0.504
	5^th^ year vs. 6^th^ year	0.24	0.029	16.579***	0.168
	5^th^ year vs. 7^th^ year	0.41	0.029	15.427***	0.272
	5^th^ year vs. 8^th^ year	0.57	0.026	14.451***	0.346
	6^th^ year vs. 7^th^ year	0.29	0.029	15.425***	0.192
	6^th^ year vs. 8^th^ year	0.45	0.026	14.449***	0.273
	7^th^ year vs. 8^th^ year	0.36	0.026	14.450***	0.219

Note. LMD (latent mean difference), SE (standardized error), * (p < 0.05), *** (p < 0.001).

## Results

### Differences in the SS development across countries, genders, and age groups

#### Differences across countries.

**Fig 2:**
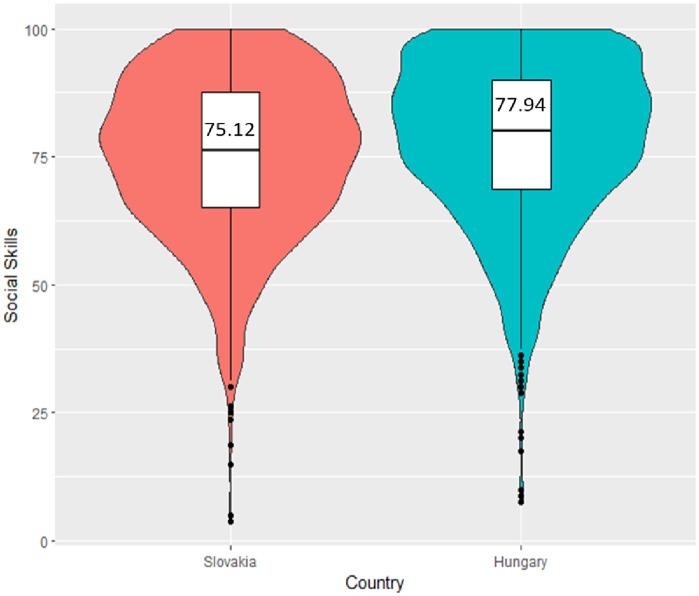
Comparison of SS development between countries.

To examine the relative progression of SS in different countries, the independent samples t-test was utilized within the R statistical software (with the ggplot2 and ggdist packages). The findings revealed a statistically significant divergence between Hungary (M = 77.94, SD = 16.12) and Slovakia (M = 75.12, SD = 15.70) in the context of SS development. This indicates that students from Hungary exhibit a superior level of SS development compared to their counterparts from Slovakia (Fig 2).

#### Differences across genders.

**Fig 3:**
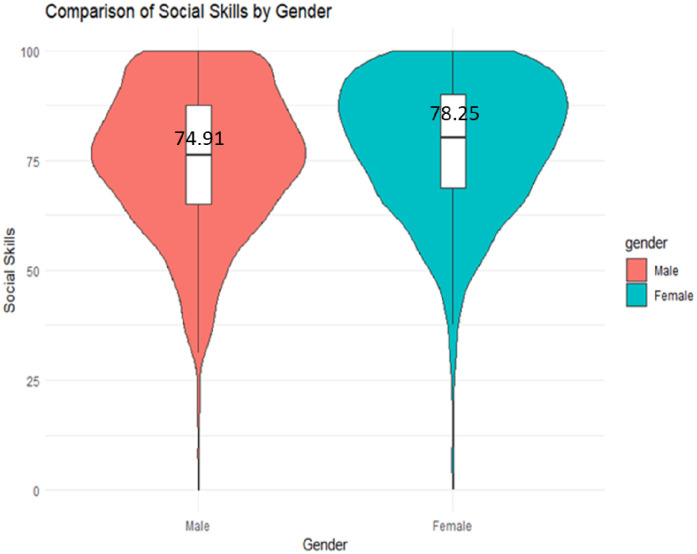
Comparison of SS development between genders.

Then, a gender-based comparison of children’s SS development was conducted. The study employed the independent samples t-test (with ggplot2 and stats packages in R) to assess gender differences in this regard. The results revealed that female students (M = 78.25, SD = 12.52) exhibited better SS development than their male counterparts (M = 74.91, SD = 16.42). This suggests that female students have a higher level of SS development compared to male students (Fig 3).

**Fig 4:**
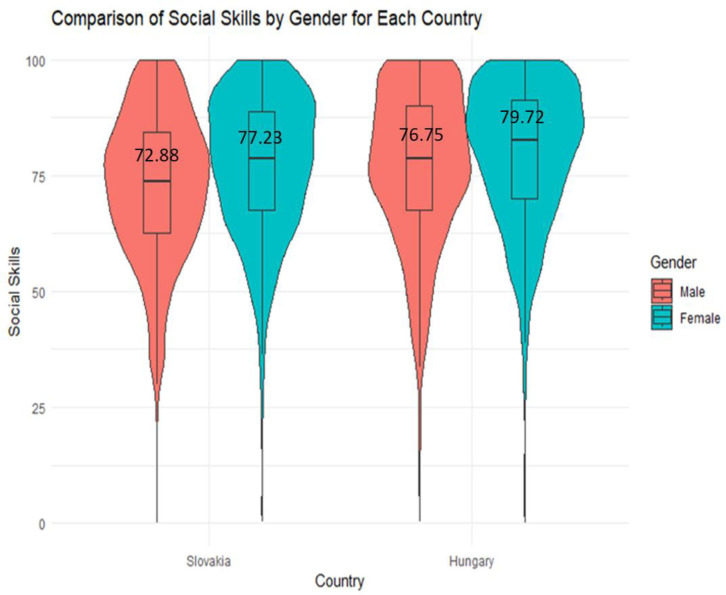
Comparison of SS development between genders for both countries.

Then, separate independent sample t-tests were conducted to compare the development of SS between genders in both Slovakia and Hungary. In the Slovakian context, the analysis revealed a statistically significant difference (***p < 0.001) between genders, indicating that girls exhibit more advanced SS (M = 77.23, SD = 15.16) compared to boys (M = 72.88, SD = 15.96). Similarly, in the Hungarian context, a significant difference between genders was observed. Here, girls displayed greater SS (M = 79.72, SD = 15.21) than boys (M = 76.75, SD = 16.61). In essence, this means that there are noteworthy disparities in social skill development between genders in both Slovakia and Hungary. In both countries, girls, on average, exhibit a higher level of SS compared to boys, and these differences are statistically significant (Fig 4).

#### Differences across age groups.

**Fig 5:**
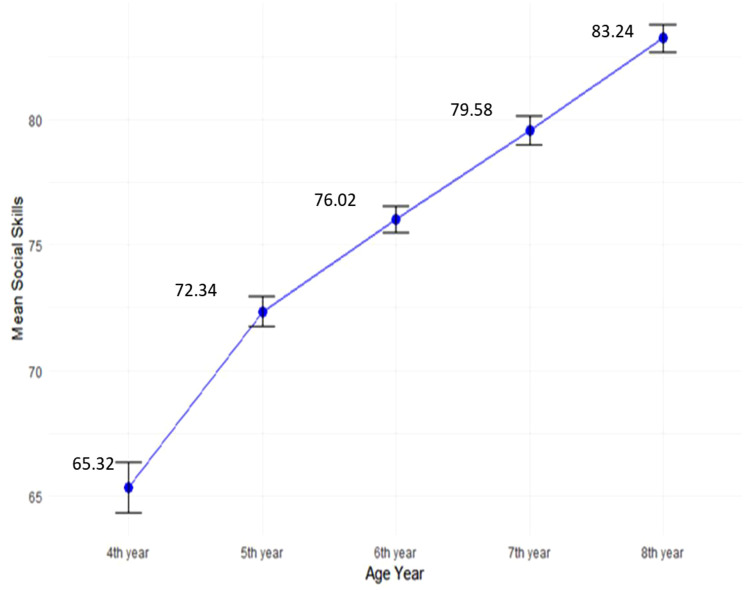
Comparison of SS development across age groups.

In the comparison of SS differences across five different age groups (4^th^, 5^th^, 6^th^, 7^th^, and 8^th^ years), the one-way analysis of variance (ANOVA) was employed by the ‘stats’ package of R. The initial examination focused on comparing SS development across all age groups within the entire sample. The results revealed statistically significant distinctions among the age groups in terms of their SS development as indicated by a significant F-statistic (F (4, 3045) = 87.62, ***p < 0.001). Specially, students in the 4^th^ grade exhibited the lowest level of SS development (M = 65.32, SD = 16.96), followed sequentially by 5^th^ years age group (M = 72.34, SD = 15.47), 6^th^ year (M = 76.02, SD = 15.28), 7^th^ year (M = 79.58, SD = 15.40), and 8^th^ year (M = 83.24, SD = 13.79), as illustrated in Fig 5.

Furthermore, the study conducted a comparative analysis involving these five groups in both countries. Initially, the significance of Levene’s statistics was evaluated to ascertain whether the assumption of equal variance was held in both countries. In Slovakia, a non-significant result (p > 0.05) was obtained, indicating the absence of a violation of the equal variance assumption. Similarly, in Hungary, the findings also showed a non-significant result (p > 0.05), signifying that there was no breach of the equal variance assumption.

**Fig 6:**
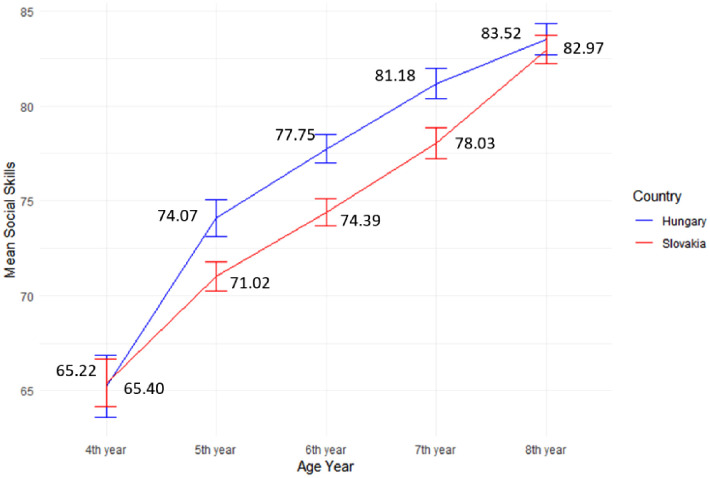
Comparison of SS development across age groups for both countries.

Subsequently, in Slovakia, the study detected significant differences among the five age groups, as indicated by an F-statistic of F(4, 1604) = 48.72, accompanied by a highly significant p-value (***p < 0.001). The analysis revealed a progressive trend in SS, with the lowest age group (4^th^ year) displaying the lowest values (M = 65.40, SD = 16.03), and the highest age group (8^th^ year) exhibiting the highest values (M = 82.97, SD = 13.19). Similarly, in Hungary, we identified a significant difference among the age groups. This ranged from the lowest values (M = 65.22, SD = 18.16) in the lowest age group (4^th^ year) to the highest values (M = 83.52, SD = 14.40) in the highest age group (8^th^ year), with F (4, 1436) = 39.16, and ***p < 0.001 (Fig 6).

### Predicting effects of background variables on SS

**Fig 7:**
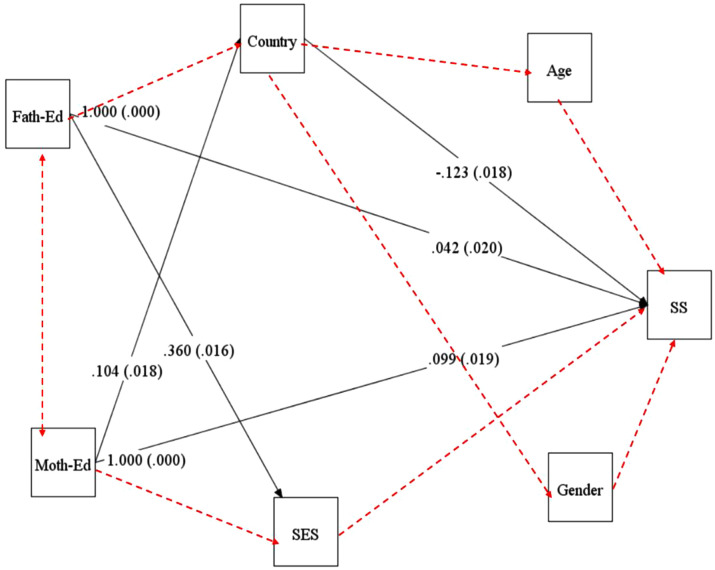
Impacts of background variables on the SS development. *Note.* SES (socio-economic status), SS (SS), Fath-Ed (father’s education), Moth-Ed (mother’s education), dotted lines represent the non-significant effects, solid lines represent the significant effects.

A comprehensive path analysis was conducted using the entire data of the sample to examine how certain background variables (country, gender, age, father’s education, mother’s education, and socioeconomic status) impact the development of students’ SS. The model fit values for this path analysis are consistent with the recommended values by Lipscomb et al. [3]. Specifically, the model fits are; χ2 = 52.6, df = 8, CFI = 0.98, TLI = 0.93, RMSEA = 0.04, SRMR = 0.05. Among these variables, only three made a significant contribution to predicting SS development: country (*β *= −0.123, Standard error = 0.018, ***p < 0.001), father’s education (*β *= 0.042, Standard error = 0.020, ***p < 0.001), and mother’s education (*β *= 0.099, Standard error = 0.019, ***p < 0.001) (Fig 7). Other variables such as gender, age levels, and socio-economic status did not have a significant impact on the overall children’s SS development.

Then, the study implemented systematic approaches specific to each country (Slovakia and Hungary) to investigate the impact of these variables (gender and age levels) on children’s SS. The study first took measures to control for family background variables, such as parental education, to ensure comprehensive analyses of these factors (gender and age levels).

### Prediction about the SS development in Slovakia

In the Slovak context, the study categorizes the factors into five distinct groups to assess both individual and combined effects. These factors include ‘gender, age, mother’s education, father’s education, and socio-economic status.’ The analysis began by considering the ‘gender’ variable in isolation within the first block and subsequently incorporating the other variables one by one over the next four blocks. The results shed light on how these background variables, either individually or in combination, affect the development of SS.

Specifically, Model 1 reveals that when the study exclusively considers the ‘gender’ variable, it accounts for approximately 11% of the variation in SS. In subsequent models – Model 2 (12.8%), Model 3 (12.9%), Model 4 (13%), and Model 5 (13%) – a gradual improvement was observed to predict students’ skills with the inclusion of additional variables. Model 2 highlights that students’ gender and age levels contribute the most to predicting their SS development, with an adjusted R-squared value of 0.128. It indicates that this model explained 12.8% of the variance in the SS development.

The robustness of these findings is corroborated by the ANOVA table stemming from the hierarchical regression analysis, which shows that all models significantly predict SS. Specifically, for Model 1 F(1, 1607) = 31.48, p < .001; for Model 2, F(2, 1606) = 118.35, p < .001; for Model 3, F(3, 1605) = 79.45, p < .001; for Model 4 F(4, 1604) = 59.71, p < .001; and for Model 5, F(5, 1603) = 47.92, p < .001. The values in Table 5, referred to as Beta weights (*β*), quantify the extent to which these background variables predict students’ SS. Among all background variables, it was found that gender and age levels of students have highly contributed to the prediction of their SS development.

**Table 5:** Hierarchical multiple linear regression analysis (Slovakia).

Model		Un-std. coef.		Std. coef.	Sig	Zero-order	Collinearity statistics	
		B	SE	*β*		Tolerance	VIF
**1**	**Gender**	4.35	0.77	0.13	<.001	0.14	1.000	1.000
**2**	**Gender**	4.56	0.73	0.14	<.001	0.14	1.000	1.000
	**Age**	4.15	0.30	0.33	<.001	0.33	1.000	1.000
**3**	**Gender**	4.53	0.73	0.14	<.001	0.14	0.998	1.002
	**Age**	4.17	0.29	0.33	<.001	0.33	0.986	1.015
	**Mother’s education**	0.70	0.55	0.03	>.05	0.02	0.996	1.005
**4**	**Gender**	4.53	0.73	0.14	<.001	0.14	0.998	1.002
	**Age**	4.17	0.30	0.33	<.001	0.33	0.996	1.004
	**Mother’s education**	1.02	0.04	0.04	>.05	0.02	0.618	1.617
	**Father’s education**	−0.59	−0.02	−0.02	>.05	−0.00	0.619	1.615
**5**	**Gender**	4.51	0.73	0.14	<.001	0.14	0.998	1.002
	**Age**	4.14	0.30	0.33	<.001	0.33	0.986	1.014
	**Mother’s education**	1.15	0.71	0.04	>.05	0.02	0.592	1.688
	**Father’s education**	−0.48	0.79	−0.01	>.05	−0.01	0.607	1.648
	**Socio-economic status**	−0.73	0.83	−0.02	>.05	−0.05	0.848	1.180

Note: dependent variable (SS)

### Prediction about the SS development in Hungary

In the Hungarian context, the results from Model 1 similarly reveal that when the study exclusively considers the ‘age’ variable, it explains approximately 10.2% of the observed changes in SS. In subsequent models – Model 2 (10.5%), Model 3 (10.6%), Model 4 (10.6%), and Model 5 (10.6%) – it was observed how the inclusion of other variables gradually improves the ability to predict students’ skills.

These findings are further substantiated by the ANOVA derived from our hierarchical regression analysis, demonstrating that all models effectively predict SS. Specifically, for Model 1, F(1, 1439) = 11.77, p < .01; for Model 2, F(2, 1438) = 85.81, p < .001; for Model 3, F(3, 1437) = 57.17, p < .001; for Model 4, F(4, 1436) = 43.55, p < .001; and for Model 5, F(5, 1435) = 34.97, p < .001. The values presented in Table 6, referred to as Beta weights (*β*), indicate the degree to which background variables contribute to the prediction of students’ SS. Among these variables, it is noteworthy that gender and age play the most significant roles in predicting students’ SS development.

**Table 6:** Hierarchical multiple linear regression analysis (Hungary).

Model		Un-std. coef.		Std. coef.	Sig	Zero-order	Collinearity statistics	
		B	SE	*β*		Tolerance	VIF
**1**	**Gender**	2.96	0.86	0.09	<.001	0.09	1.000	1.000
**2**	**Gender**	4.19	0.82	0.12	<.001	0.09	0.986	1.014
	**Age**	4.14	0.32	0.31	<.01	0.30	0.986	1.014
**3**	**Gender**	4.20	0.82	0.12	<.001	0.09	0.984	1.016
	**Age**	4.14	0.33	0.31	<.001	0.30	0.979	1.021
	**Mother’s education**	−0.10	0.61	−0.00	>.05	−0.02	0.990	1.010
**4**	**Gender**	4.17	0.82	0.13	<.001	0.09	0.984	1.017
	**Age**	4.13	0.33	0.31	<.001	0.30	0.979	1.022
	**Mother’s education**	−0.88	0.78	−0.03	>.05	−0.02	0.607	1.646
	**Father’s education**	1.34	0.84	0.05	>.05	0.02	0.611	1.637
**5**	**Gender**	4.18	0.82	0.13	<.001	0.09	0.984	1.017
	**Age**	4.14	0.33	0.31	<.001	0.30	0.978	1.022
	**Mother’s education**	−1.04	0.81	−0.04	>.05	−0.02	0.573	1.746
	**Father’s education**	1.20	0.86	0.04	>.05	0.02	0.589	1.699
	**Socio-economic status**	0.74	0.89	0.02	>.05	0.00	0.792	1.262

Note: dependent variable (SS).

## Discussion

This study investigated the MI of the SS assessment of the DIFER test, and LMD across the country (Hungary and Slovakia), gender (male and female), and age (4^th^, 5^th^, 6^th^, 7^th^, and 8^th^ years). First, based on the findings from the measurement invariance across countries, genders, and age groups, it was found that the measurement invariance of the SS test across these different groups had been established. This indicates that the SS test is a reliable and valid tool for assessing school readiness among Hungarian-speaking children from Hungary and Slovakia, across genders and age groups. Several recent studies have reported similar findings regarding the invariance of SS across different populations. For example, a study by Zhu et al. [8] pointed out the measurement invariance across two countries of China and Japan, indicating that the SS assessment is reliable and valid for measuring preschool children’s school readiness. These findings are consistent with previous research that has shown SS to be an important factor in school readiness and academic success [16]. Martín-Puga et al. [29] also showed a measurement invariance across genders and ages. The establishment of measurement invariance is essential for ensuring that test scores are comparable across different groups, which is necessary for making accurate comparisons and drawing valid conclusions [1,23]. Then, this study also investigated the latent mean differences in SS assessment for children across countries, genders, and ages. Our results showed that there were significant differences in the latent ability levels between countries, genders, and age groups. Specifically, young children from Hungary had significantly higher latent ability levels than those from Slovakia. This finding is consistent with previous research on cultural differences in SS development, which suggests that children from different cultures may have different levels of social competence due to varying cultural norms and values [19].

In terms of gender differences, the findings showed that female students had higher latent ability levels than male students. This is in line with previous research on gender differences in SS, which has consistently found that girls tend to have higher levels of social competence than boys [22]. This could be due to a variety of factors, including differences in socialization practices, cognitive processing, and interpersonal communication styles. Finally, the results also showed that the older age groups had higher latent ability levels than the younger age groups. This is consistent with previous research on developmental changes in SS, which suggests that social competence tends to improve with age due to factors such as increased cognitive and emotional maturity, expanded social networks, and more opportunities for social interactions [37].

This study also explored the complex characteristics of children’s SS development to uncover variations across different contexts and identify predictors contributing to their SS development. We examined the influence of the national context (country) on SS development. The findings illuminated a significant disparity between Hungary and Slovakia. Notably, children in Hungary exhibited a significantly higher level of SS development than those from Slovakia. This divergence aligns with other studies [8,21] that show the profound impact of the national environment on shaping children’s SS. Moreover, it is essential to consider that these disparities could result from a multitude of factors beyond just the national environment. Children’s gender differences, age groups, socio-economic status, and their parents’ education levels may all contribute to this divide, emphasizing the complex nature of SS development.

Moving to gender-based differences, the analysis revealed a consistent pattern. Across both countries, female students displayed more advanced SS compared to their male peers. The studies reviewed above [22,23] are also consistent with our finding of this gender difference. However, it is crucial to explore the underlying mechanisms driving these disparities. Beyond peer cooperation or social tasks, socialization processes and societal expectations may all play integral roles. Understanding the nuances of gender-related SS development could pave the way for more targeted interventions to bridge this gender gap. Age emerged as another influential factor in our study. As children advanced in age, their SS tended to improve, indicating an age-related progression in SS. This pattern held true in both countries, emphasizing the ongoing developmental nature of SS. This aligns with the widely accepted notion that SS evolves and matures over time [21]. Nevertheless, it is imperative to acknowledge that individual variations exist within each age group. Exploring the factors contributing to these variations, such as parenting styles and peer dynamics, can provide a richer understanding of age-related trends.

Parental education played an essential role in predicting children’s SS development. Specifically, children with highly educated parents demonstrated higher SS. Several research findings show the importance of parental education in shaping children’s SS development [24,25,41–43]. Surprisingly, socioeconomic status did not emerge as a significant predictor in our analysis, which is the opposite of the result of the referenced study [25]. The possible reason may be that within the scope of the current study, other factors, such as parental education and country, had more substantial effects on SS development. Moreover, exploring the potential mediating factors between socio-economic status and SS can shed light on the underlying dynamics.

As for practical implications, this study contributes to the growing body of literature on school readiness and highlights the importance of SS in this process. This study averred the importance of SS in children’s development and their cultural variations. The observed differences between Slovakia and Hungary suggest that culture influences the acquisition and expression of SS. Educators, policymakers, and parents should consider cultural context when designing interventions and programs to enhance SS. Age differences in social skills development highlight the need for age-appropriate interventions and targeted support. Maternal education’s role in social skill development emphasizes the influence of family factors. This knowledge can guide efforts to promote SS, considering sociocultural context and involving parents in promoting positive social development.

There are some limitations in the study. Firstly, the study primarily relied on quantitative data, which may not capture the full depth and details of children’s SS development. Moreover, in this digital age, the relationships between digital technologies and children’s social skills development were not explored in the current study. Therefore, future studies were recommended to explore the importance of digital media in children’s social skills development. Additionally, the cross-sectional nature of the research design restricts the authors’ ability to establish causal relationships and understand the long-term trajectory of SS development.

### Conclusion

To conclude, this study has been conducted to investigate the differences in children’s SS development across countries, genders, and age groups. Moreover, predicting variables on their SS development were also explored. Before comparing children’s SS development, the MI of the SS assessment instrument was confirmed across the different groups of countries, genders, and age groups. The findings confirmed the reliability and consistency of the SS assessment test across diverse groups of Hungarian-speaking children, demonstrating MI across all full scalar levels (configural, metric, scalar) and significant LMD. Moreover, the comparative analyses revealed significant differences in children’s SS development between Slovakia and Hungary, with Hungarian children demonstrating superior SS levels. Gender emerged as a significant factor, with girls exhibiting better SS progress compared to their boy counterparts. Additionally, age played a crucial role, as older children within their respective age groups displayed significantly higher SS levels than younger children. Importantly, while parental education emerged as a significant predictor of SS development across the entire sample, the examination of each country separately revealed distinct variations, highlighting the complex interplay of social and cultural factors. Therefore, this study contributes valuable insights for educators and researchers, emphasizing the importance of contextual factors in the assessment of children’s SS development.
